# Children and Ultraviolet Radiation

**DOI:** 10.3390/children9040537

**Published:** 2022-04-11

**Authors:** Olaf Gefeller, Katharina Diehl

**Affiliations:** Department of Medical Informatics, Biometry and Epidemiology, Friedrich-Alexander-Universität Erlangen-Nürnberg (FAU), 91054 Erlangen, Germany; katharina.diehl@fau.de

Solar radiation is one of the driving forces for life on earth. We are warmed by the infrared part of its electromagnetic spectrum, and we perceive our environment with eyes responding to the visible part of the spectrum. Visible light is also an essential component of photosynthesis, the process used by plants and other organisms to convert light energy into chemical energy that can later be released to fuel the organism’s activities. In addition to these positive and virtually essential properties for life on our planet, solar radiation also has deleterious effects on biological systems. The negative consequences of solar radiation are due primarily to radiation within the ultraviolet (UV) part of the electromagnetic spectrum [1]. The UV region of the spectrum is confined to wavelengths between 100 nm and 400 nm, and subdivided into three bands termed UVA (400–315 nm), UVB (315–280 nm) and UVC (280–100 nm). The cutoff values for the subdivisions are arbitrary and differ somewhat depending on the discipline involved. Both the spectrum and the intensity of terrestrial UV radiation varies with the elevation of the sun above the horizon, which is measured by the solar zenith angle (SZA) defined as the angle between the local zenith (i.e., directly above the point on the ground) and the line of sight from that point to the sun [2]. The SZA as the main driver of the intensity of natural UV radiation depends on the geographical location, season and time of day. In addition to the SZA, atmospheric conditions, cloud coverage and the UV reflectance from the surface (termed albedo effect) affect the intensity of UV radiation perceived by unprotected humans being exposed to solar radiation [3].

The International Agency for Research on Cancer, an institution operated by the World Health Organization, has officially classified UV radiation as carcinogenic to humans and placed it in the group I of those carcinogens with the strongest evidence for carcinogenicity [4]. Numerous experimental and epidemiologic studies have demonstrated that UV radiation, irrespective of whether resulting from natural solar origin or from artificial sources such as tanning devices, increases the risk of different types of skin cancer, most importantly that of melanoma, the most lethal type of skin cancer [5,6]. While the risk increasing effect of UV exposure for humans is not confined to special age groups [7], children are an especially vulnerable group that needs tailored concepts for protection [8,9]. Over the first years of life, children’s skin barrier protection remains immature. As a consequence, early UV exposure induces rapidly actinic skin damage [10]. UV overexposure during childhood has also been found to be associated with higher nevus density [11]. The importance of nevi, particularly atypical nevi referred to as ‘dysplastic nevi’, in the development of melanoma has been stressed in numerous studies: Not only should nevi be considered an extremely strong marker of melanoma risk, they are frequently also precursors of melanoma, as approximately half of all melanomas do not develop de novo but arise on originally benign nevi [12]. Furthermore, the amount of UV radiation received over a lifetime is not uniformly distributed over all ages [13]. Children accumulate substantially higher doses of UV radiation through outdoor activities than adults, as they spent typically a smaller proportion of the day indoors. Finally, because of the long latency period of skin cancer, children are more susceptible than older adults to the initiation of latent harmful effects of UV radiation that manifest decades later, since older adults do not live long enough to experience the manifestation of skin cancer due to competing risks. 

In this Special Issue devoted to children and UV radiation, we have compiled five articles, all representing original research, that tackled different aspects of the topic. All articles went through rigorous peer review by ourselves and independent external reviewers, whom we want to thank for their time and effort to improve and clarify the presentation of findings in these articles. 

Two papers in this Special Issue addressed UV radiation in the preschool setting. Seidel et al. [14] described the ‘Clever in Sun and Shade for Preschools’ program (CLEVER) that combined theory-based individual and environmental interventions to reduce skin cancer risks in preschools and targeted staff members, children, and parents. The effectiveness of CLEVER was assessed in a cluster-randomized trial of high methodological quality in 24 German preschools with 273 staff members. Staff members of preschools taking part in CLEVER differed significantly from those in the control group in a number of relevant outcome measures after 12 months. The results showed that CLEVER can easily be disseminated and can strengthen sun protection for children in preschools. The second paper from the preschool setting, by Gefeller et al. [15], addressed another aspect of sun protection in preschools. Their interest was focused on the Global Solar UV index (UVI), a simple means of visualizing the intensity of UV radiation and thereby alerting people to the need for sun protection. In a survey among directors of 436 preschools in southern Germany, they observed that less than half of the directors had ever heard of the UVI, few had correct and detailed knowledge about the UVI, and only a small minority of them used UVI information to adapt sun protective measures in their preschools. They concluded that their study provided a sobering picture regarding the penetration of the UVI in German preschools.

A second focus of this Special Issue was on information provided by caregivers regarding children’s sun protection behavior. The study from Portugal by Salvado et al. [16] revealed high knowledge among caregivers regarding sun protection in children. In addition, the majority indicated that they follow international guidelines on skin cancer prevention when applying sunscreen. The German study by Görig et al. [17] showed differences in compliance with various sun protection measures. While sunscreen was the most common measure used, sunglasses were least frequently used. The prevalence of sunburn increased with age, while the prevalence of using sun protection measures decreased. Overall, use of sun protection measures was higher when caregivers perceived themselves as a role-model, which seems to be important for future prevention campaigns.

Another source of UV radiation besides natural sunlight are UV-emitting tanning devices such as sunbeds. Since 2011, use of commercial sunbeds by minors is prohibited in England. Gordon et al. [18] described that despite this legal ban for minors, there were still 62,000 children and adolescents in England who used commercial sunbeds. They suggested a ‘buy-back’ scheme which follows the example of Australia to encourage the removal of sunbeds, while financially compensating sunbed providers for the enforced changes to their business. Based on their economic analysis, such a ‘buy-back’ scheme for an estimated number of 18,000 commercial sunbeds might cost the English government upwards of GBP 55 million, but would be a one-time investment for a permanent solution to remove access for young people.

To prevent children and adolescents from the harmful effects of UV radiation, different starting points are possible. These can also be combined with each other. On the one hand, it is important to educate about the potential negative consequences of UV radiation in childhood and adolescence. Here, campaigns can be helpful to spread the knowledge from guidelines among the population [19]. On the other hand, regulatory measures, such as bans on the use of sunbeds [20] or the ‘buy-back’ scheme presented [18], can also be helpful building blocks. Interdisciplinary approaches using multifaceted strategies to tackle the challenges on the way to a conscious and healthy handling of UV radiation during childhood and adolescence are necessary.

The five papers in this Special Issue highlighted only some aspects of the much broader topic ‘children and UV radiation’. For example, experimental studies elucidating the cellular and molecular mechanisms in UV irradiated juvenile skin, clinical studies addressing the therapeutic management of childhood melanoma induced by UV radiation, and epidemiologic studies based on pediatric disease registers such as melanoma registers of adolescent cases [21] correlating disease data to environmental data regarding UV radiation were lacking in this Special Issue. These and other aspects delineating the role of UV radiation for children’s health will continue to have a place in the journal *Children*. Manuscripts describing new findings from well-designed studies are welcome—depending on the specific topic—in the new section ‘Pediatric Dermatology’ and in the section ‘Global and Public Health’ of *Children*.

## Data Availability

Not applicable.
